# High-Sensitivity Magnetic Field Sensor Based on an Optoelectronic Oscillator with a Mach–Zehnder Interferometer

**DOI:** 10.3390/s25051621

**Published:** 2025-03-06

**Authors:** Mingjian Zhu, Pufeng Gao, Shiyi Cai, Naihan Zhang, Beilei Wu, Yan Liu, Bin Yin, Muguang Wang

**Affiliations:** 1 Key Laboratory of All Optical Network and Advanced Telecommunication Network, Ministry of Education, Institute of Lightwave Technology, Beijing Jiaotong University, Beijing 100044, China; 22120199@bjtu.edu.cn (M.Z.); 22110010@bjtu.edu.cn (P.G.); 20111012@bjtu.edu.cn (S.C.); wubeilei@bjtu.edu.cn (B.W.); yanliu@bjtu.edu.cn (Y.L.); 2 China United Network Communications Group Corporation Limited, Beijing 100033, China; zhangnh12@chinaunicom.cn; 3 School of Information and Communication Engineering, Beijing University of Posts and Telecommunications, Beijing 100876, China; 4 College of Information Science and Engineering, Ocean University of China, Qingdao 266100, China; binyin@ouc.edu.cn

**Keywords:** microwave photonics, optical fiber sensing, optoelectronic oscillator, Mach–Zehnder interferometer, magnetic field measurement

## Abstract

A high-sensitivity magnetic field sensor based on an optoelectronic oscillator (OEO) with a Mach–Zehnder interferometer (MZI) is proposed and experimentally demonstrated. The magnetic field sensor consists of a fiber Mach–Zehnder interferometer, with the lower arm of the interferometer wound around a magnetostrictive transducer. Due to the magnetostrictive effect, an optical phase shift induced by magnetic field variation is generated between two orthogonal light waves transmitted in the upper and lower arms of the MZI. The polarization-dependent property of a Mach–Zehnder modulator (MZM) is utilized to transform the magnetostrictive phase shift into the phase difference between the sidebands and optical carrier, which is mapped to the oscillating frequency upon the completion of an OEO loop. High-sensitivity magnetic field sensing is achieved by observing the frequency shift of the radio frequency (RF) signal. Temperature-induced cross-sensitivity is mitigated through precise length matching of the MZI arms. In the experiment, the high magnetic field sensitivity of 6.824 MHz/mT with a range of 25 mT to 25.3 mT is achieved and the sensing accuracy measured by an electrical spectrum analyzer (ESA) at “maxhold” mode is 0.002 mT. The proposed sensing structure has excellent magnetic field detection performance and provides a solution for temperature-insensitive magnetic field detection, which would have broad application prospects.

## 1. Introduction

Magnetic field sensing is critical in applications across various sectors, including industrial automation, defense, and aerospace [1]. Magnetic field detection is valuable in engineering applications such as underwater pipeline control [2], tsunami warning systems [3], biomedical sensing [4], and urban road construction [5], where numerous sensors are needed for real-time monitoring of data changes. In industrial and environmental monitoring, magnetic sensors are essential for detecting equipment malfunctions, ensuring structural integrity, and tracking environmental changes such as magnetic anomalies caused by pollution or geological shifts. Traditional magnetic field sensors, often reliant on metal and electronic components, are prone to electromagnetic interference and thermal drift, limiting their stability and accuracy in fluctuating environments. In contrast, fiber-optic magnetic field sensors have experienced swift advancement, distinguished by their diminutive form factor, robustness, and immunity to corrosion, setting them apart from traditional sensing technologies. Their compact size, high sensitivity, and durability enable precise magnetic field detection in harsh environments. As a result, fiber-optic magnetic field sensors are increasingly adopted in cutting-edge technologies, preparing for more accurate and efficient magnetic field sensing systems in the future [6].

Fiber-based magnetic field sensors assess surrounding magnetic fields by discerning their influence on various attributes of the light signal, including phase changes, intensity fluctuations, polarization state modifications, and wavelength variations. A multitude of magnetic field sensing methodologies have been conceptualized, each employing distinct optical fiber structures, including the fiber modal interferometer [7,8], surface plasmon resonance [9], fiber Bragg grating [10], and fiber Fabry–Perot cavity [11]. Fiber-optic magnetic field sensors frequently integrate materials with magnetic sensitivity, such as magnetic fluids and magnetostrictive materials, to facilitate the detection and measurement of magnetic fields. Conventionally, the wavelength demodulation in magnetic field sensors is accomplished through the utilization of an optical spectrum analyzer (OSA). However, the inherent limitations of OSAs in terms of scanning speed and resolution significantly impact the demodulation accuracy and temporal response of fiber-optic magnetic field sensors, thereby hindering their potential in high-resolution and fast-response sensing applications. Specifically, the scanning speed of OSAs restricts the system’s ability to capture rapidly changing magnetic field signals, while their limited resolution may lead to the oversight of minor wavelength variations, consequently reducing the sensor’s measurement precision. Therefore, the development of an innovative demodulation technology with enhanced speed and resolution is of paramount importance. Such technology must not only be capable of processing rapidly changing signals in real time but also possess high sensitivity to detect subtle wavelength variations, meeting the requirements of modern high-precision sensing measurements. By overcoming the limitations of existing technologies, this new demodulation approach has the potential to expand the applications of fiber-optic magnetic field sensors in industrial inspection, medical diagnostics, scientific research, and beyond.

Microwave photonics (MWP), as a novel interdisciplinary technology, combines the advantages of microwave and photonic technologies to enable rapid and high-resolution measurement of external environmental parameters by mapping changes in optical signals to microwave signals [12]. The optoelectronic oscillator functions as a resonant cavity in microwave photonic systems, producing microwave signals characterized by low phase noise and superior quality [13,14]. Recent research has explored OEO-based sensing for parameters such as strain, refractive index, and temperature [15,16,17,18,19]. In the realm of magnetic field detection, Wu et al. introduced the demodulation scheme that combines magnetostrictive alloys with fiber Bragg grating Fabry–Perot filters, achieving magnetic field sensitivity of 384 MHz/mT, though its complex fabrication process increases costs [12]. Feng et al. developed a temperature-compensated magnetic field sensor, utilizing principles of an OEO, which addresses temperature crosstalk issues by cascading magnetostrictive alloy fiber Bragg gratings with standard FBGs, achieving the sensitivity of 2.5 kHz/mT [20]. The FBG-based sensors generally have lower sensitivity than FBG-FP designs, despite their simpler structure. Sun et al. have developed a magnetic field sensor utilizing a dual-ring OEO and etched fiber Bragg gratings, achieving the sensitivity of 163 Hz/mT, though it faces a temperature crosstalk challenge [21]. Compared with FBG and FBG-FP structures, the OEO magnetic field sensor based on the Mach–Zehnder interferometer structure reduces temperature crosstalk by simply matching the lengths of the two arms. This design not only avoids the need for additional cascaded components, but also significantly reduces manufacturing costs and system complexity. In terms of ease of implementation, the assembly and debugging process of the MZI structure is relatively simple, suitable for large-scale production and rapid deployment. In addition, due to the variability of the number of turns of the fiber wrapped around the magnetostrictive transducer, it provides a certain degree of scalability for the system.

This paper presents a high-sensitivity magnetic field sensing structure using an MZI coupled with an OEO, where the lower arm is wrapped around a magnetostrictive transducer. This transducer features a rectangular block of giant magnetostrictive material (GMM) clamped by two supporting arms tightly wrapped with multiple turns of fiber. When affected by a magnetic field, the GMM undergoes deformation, which is conveyed via the supporting arm to the optical fiber coiled around it. The deformation alters the optical path length within the MZI, resulting in a shift of the relative phase of the light waves. Utilizing the polarization-dependent property of a lithium niobate (LiNbO_3_) modulator, the magnetostrictive effect-induced phase shift is transformed into a phase difference between the sideband and the optical carrier, both of which maintain orthogonal polarization states. In the OEO loop, the phase difference is then transformed into oscillation frequency of the RF signal. Thus, the frequency shift of the RF signal can reflect the variation in the magnetic field. Furthermore, temperature crosstalk issues can be mitigated by matching the fiber length in the two arms of the MZI.

## 2. Operation Principle

**Figure 1:**
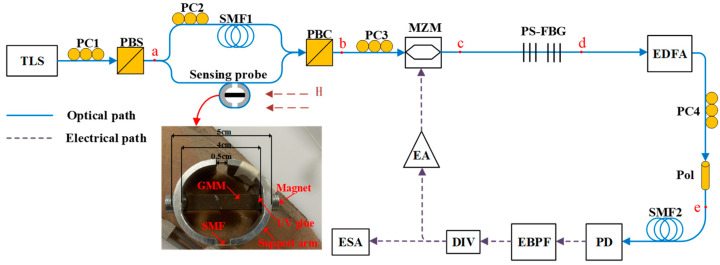
Schematic layout of an OEO-based magnetic field sensing system with enhanced sensitivity. Points a–e: the optical signals output from the PBS, PBC, MZM, PS-FBG and Pol, respectively.

**Figure 2:**
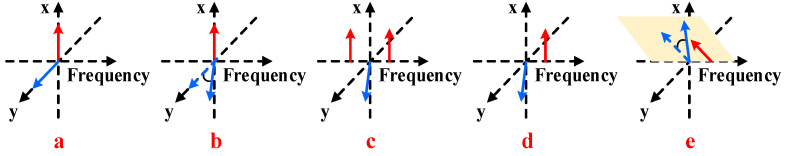
Optical spectral transformation of OEO. (**a**–**e**) the optical spectra of the signals emitted from the PBS, PBC, MZM, PS-FBG and Pol, respectively; Red and blue arrows: two optical signals with specific amplitudes and polarization states transmitted along the fiber.

The magnetic field sensing system based on an OEO is illustrated schematically in Figure 1. Linearly polarized light emitted from a tunable laser source (TLS) is divided into two optical signals via a polarization controller (PC1) and polarization beam splitter (PBS). The PC1 is calibrated so that the polarization angle of the incoming light is set at 45° relative to the polarization axis of the PBS, thereby producing two light waves with orthogonal x and y polarizations and identical amplitudes, which propagate through a single-mode fiber (SMF) starting from point a, as depicted in Figure 2a.

As x and y polarized lights propagate along the lower and upper arms of the MZI, the magnetostrictive effect introduces an optical phase shift caused by the magnetic field between the two orthogonal light waves. The PC2 is placed in the upper arm to maintain the orthogonality of the lights transmitted through the two arms of the MZI. The x and y polarized light beams are merged using a polarization beam combiner (PBC) and subsequently directed to the MZM through a PC3, aligning the x-polarized light with the principal axis of the MZM. Owing to the polarization-dependent property of LiNbO_3_ crystal, the y-polarized light traverses the MZM without undergoing modulation. A phase-shifted fiber Bragg grating (PS-FBG) is then utilized to selectively eliminate −1st-order sideband from the light spectrum.

Two orthogonally polarized light waves are intensified by an erbium-doped fiber amplifier (EDFA) and then interfered at a polarizer (Pol) with the assistance of a PC4. After passing through a photodetector (PD), the generated interference light is transformed into an RF signal, which is subsequently filtered through an electrical bandpass filter (EBPF). The signal is then split by a power divider, with one branch being monitored in real time using an ESA to analyze the spectral characteristics. The other branch is enlarged by an electrical amplifier (EA) and fed back into the MZM to establish the OEO feedback loop.

Figure 2 illustrates the spectral variations in the OEO system at different processing stages, where x and y labels represent the polarization axes. Point b depicts the spectrum after passing through the MZI, where the phase difference between its two arms exhibits a linear correlation with the external magnetic field due to the magnetostrictive effect. Point c represents the modulated spectrum output from the MZM, reflecting the precise electro-optic modulation effect. Point d highlights the filtered spectrum after the PS-FBG, which serves as an optical bandpass filter (OBPF). Point e presents the spectrum output after passing through the Pol, ensuring optimal signal alignment via polarization state optimization. Together, these points demonstrate the intricate interplay of optical and electrical components in achieving high-performance magnetic field sensing.

**Figure 3:**
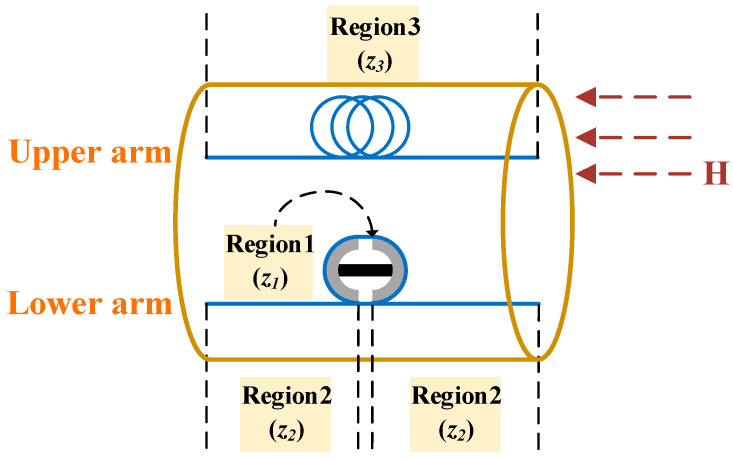
The schematic of the fiber’s various regions within the solenoid.

The design of the sensing component in the temperature-compensated magnetic field sensor is depicted in Figure 3. Inside the solenoid, optical fibers utilized for detecting the magnetic field are categorized into distinct regions. The fibers in Region 2 and 3 remain unmodified and are only sensitive to ambient temperature changes, serving as a reference for temperature compensation, with no response to variations in the magnetic field. In contrast, the fiber in Region 1 is wound around a magnetostrictive transducer made of the GMM. When exposed to the magnetic field, the GMM undergoes magnetostrictive deformation, which induces strain in the fiber wound around two supporting arms and altering its length and refractive index. Consequently, the optical fiber situated in Region 1 exhibits sensitivity to variations in both magnetic field and temperature. When the ambient temperature or magnetic field in the Region *i* (where *i* = 1, 2, 3) changes, the length of the fiber $z_{i}$ and effective refractive index $n_{i}$ will undergo corresponding changes, which can be represented as [22]:

(1) $$\begin{array}{l} \left\{\begin{array}{l} \Delta z_{1}=\left[\alpha_{F}\Delta T+\left(\frac{\pi l_{Al}}{\pi d+2l_{1}}\alpha_{Al}\Delta T+\frac{2l_{GMM}}{\pi d+2l_{1}}\left(\alpha_{M}\Delta T+k\Delta B\right)-\alpha_{F}\Delta T\right)\right]\cdot z_{1}\\ \Delta z_{2}=\alpha_{F}\cdot \Delta T\cdot z_{2}\\ \Delta z_{3}=\alpha_{F}\cdot \Delta T\cdot z_{3} \end{array}\right.\\ \left\{\begin{array}{l} n_{1}=n_{2}=n_{3}=n\\ \Delta n_{1}=n\cdot \zeta \cdot \Delta T-n\cdot P_{e}\left[\frac{\pi l_{Al}}{\pi d+2l_{1}}\alpha_{Al}\Delta T+\frac{2l_{GMM}}{\pi d+2l_{1}}\left(\alpha_{M}\Delta T+k\Delta B\right)-\alpha_{F}\Delta T\right]\\ \Delta n_{2}=\Delta n_{3}=n\cdot \zeta \cdot \Delta T \end{array}\right. \end{array}$$

where $\Delta z_{i}$, $\Delta n_{i}$ are length and refractive index changes of the fibers in Region *i*, ΔB, ΔT are the variations of the magnetic field and temperature, and *k* is a coefficient proportional to the magnetostrictive constant of the GMM (assuming that the magnetostrictive material exhibits linear behavior under the applied magnetic field, which is effective for the magnetic field range used in our experiment). ζ is the thermo-optic coefficient. It is worth noting that ‘n⋅ζ⋅ΔT’ represents the refractive index change caused by the temperature effect on the fiber itself in each region. d, $l_{1}$ denote the outer diameter and spacing of the two supporting arms, $l_{GMM}$, $l_{Al}$ represent the length of the GMM and the total thickness of the two aluminum supporting arms, and $\alpha_{F}$, $\alpha_{M}$, $\alpha_{Al}$ are the thermal expansion coefficients of the fiber, GMM, and supporting arms. It is noteworthy that ‘$\alpha_{F}\Delta T$’, ‘$\alpha_{Al}\Delta T$’ and ‘$\alpha_{M}\Delta T$’ respectively represents the fiber, aluminum material, and GMM caused by their own temperature effects, and ‘kΔB’ is the strain generated by the GMM under the influence of the external magnetic field. Therefore, through transformation, the external strain exerted on the fiber wound around the magnetostrictive transducer, which is affected by both temperature and magnetic field, can be expressed as ‘$\frac{\pi l_{Al}}{\pi d+2l_{1}}\alpha_{Al}\Delta T+\frac{2l_{GMM}}{\pi d+2l_{1}}\left(\alpha_{M}\Delta T+k\Delta B\right)-\alpha_{F}\Delta T$’. $P_{e}$ is the effective elastic-optic coefficient of the fiber. Given the thermal strain of the optical fiber itself and the external strain imposed by the magnetostrictive transducer on the fiber within the sensing component, we can deduce the variations in its length and refractive index, as shown in Equation (1).

In the solenoid, the phase difference $\Delta \varphi_{LU}$ between two orthogonal light waves, induced by variations in magnetic field and temperature, can be written as:

(2) $$\begin{array}{cc} \Delta \varphi_{LU}= & \varphi_{lower}-\varphi_{upper}\\ = & k_{0}[(\Delta n_{1}z_{1}+n\Delta z_{1}+\Delta n_{2}z_{2}+n\Delta z_{2})-(\Delta n_{3}z_{3}+n\Delta z_{3})]\\ = & \left\{\left(\zeta +\alpha_{F}\right)\left(z_{2}-z_{3}\right)\right.\\ + & \left.\left[\zeta +\frac{\pi l_{Al}}{\pi d+2l_{1}}\alpha_{Al}+\frac{2l_{GMM}}{\pi d+2l_{1}}\alpha_{M}-P_{e}\left(\frac{\pi l_{Al}}{\pi d+2l_{1}}\alpha_{Al}+\frac{2l_{GMM}}{\pi d+2l_{1}}\alpha_{M}-\alpha_{F}\right)\right]z_{1}\right\}n\cdot \Delta T\cdot k_{0}\\ + & \left(1-P_{e}\right)\cdot k_{0}n\cdot \frac{2l_{GMM}}{\pi d+2l_{1}}\cdot k\Delta B\cdot z_{1} \end{array}$$

where $\varphi_{upper}$, $\varphi_{lower}$ are the phase changes of light signals in two arms of the MZI induced by magnetic field and temperature variations, while $k_{0}$ is the propagation constant in a vacuum. As evident from Equation (2), if the fiber length utilized to construct the sensing probe ($z_{1}$) is established, the component associated with ΔT can be nullified by choosing suitable values for $z_{2}$ and $z_{3}$. Consequently, the impact of temperature on the sensing component can, in theory, be entirely eliminated. This temperature compensation design relies on the precise matching of the optical fiber lengths in the two arms of the MZI.

In the OEO loop, when light propagates through the MZI to the MZM, it is necessary to adjust the DC bias of the MZM to the minimum transmission point in order to achieve carrier-suppressed double sideband (CS-DSB) modulation. The modulation method requires a fixed phase difference of ‘$\left(2k+1\right)\pi$’ to be introduced between the upper and lower arms of the MZM to ensure that the optical carrier is suppressed while the ±1st-order sidebands are enhanced, which is critical for high-quality beat signal generation in the OEO system. At point c, owing to the polarization-dependent property of the MZM, ±1st-order sidebands and an optical carrier with orthogonal polarization relationships are generated, as illustrated in Figure 2c. The phase shift between the optical carrier and sidebands corresponds to the magnetostrictive phase difference $\Delta \varphi_{LU}$ in the two arms of the MZI. At point d, +1st-order sideband and the optical carrier, which possess mutually orthogonal polarization relationships, are retained after being filtered by a PS-FBG. If the PC4 adjusts the polarization direction of its emitted light at a 45° angle to the principal axis of Pol, the interference signal at point e can be written as:

(3) $$\begin{array}{cc} E_{out}\left(t\right) & \;\propto E_{c}\exp\left[j\omega_{c}\left(t+\tau_{s}\right)+j\varphi_{lower}\right]\\ & \;+E_{c}J_{1}\left(\beta \right)\exp\left[j\left(\omega_{c}+\omega_{e}\right)\left(t+\tau_{s}\right)+j\varphi_{upper}\right] \end{array}$$

where $\omega_{c}$, $E_{c}$ are the angular frequency and amplitude of the optical carrier, $J_{n}$ denotes the nth-order Bessel function, $\beta =\pi V_{e}/V_{\pi }$ is the modulation index, $V_{\pi }$ is the half-wave voltage of the MZM, $\omega_{e}$, $V_{e}$ are the angular frequency and amplitude of the RF signal, and $\tau_{s}=n_{s}L_{s}/c$ is the time delay of SMF2, where $L_{s}$ and $n_{s}$ are the length and refractive index of SMF2, respectively, and c is the velocity of light in a vacuum. The output photocurrent of the PD can be written as:

(4) $$i_{PD}\left(t\right)\propto {\left|E_{c}\right|}^{2}J_{1}\left(\beta \right)\cos\left[\omega_{e}t+\omega_{e}\tau_{s}-\left(\varphi_{lower}-\varphi_{upper}\right)\right]$$

At the PD output, the magnetostrictive phase difference $\Delta \varphi_{LU}$ is transformed into the phase shift of the RF signal as indicated by Equation (4).

Upon closing the OEO loop, the RF signal must satisfy the phase matching condition, which means that after the light wave propagates through a complete round-trip path, the accumulated phase difference should be an integer multiple of 2π. Only under this condition can each cycle of the frequency component be coherently superimposed at any given moment, rather than undergoing destructive interference. Therefore, the frequency at the oscillation peak can be expressed as:

(5) $$2\pi f_{e}t+2\pi f_{e}\tau_{s}-\Delta \varphi_{LU}=2\pi \gamma$$

where γ is an integer, $f_{e}$ is the frequency of the oscillation signal of the OEO, and $t=n_{s}L_{OEO}/c$ is the time delay of the OEO, where $L_{OEO}$ is the loop length of the OEO. Based on Equations (2) and (5), the shift in microwave frequency caused by the magnetic field can be written as:

(6) $$\begin{array}{cc} \Delta f_{e}= & \frac{c}{2\pi n_{s}L_{OEO}}\cdot \frac{\Delta \left(\Delta \varphi_{LU}\right)}{\Delta B}\cdot \Delta B\\ = & \frac{c}{2\pi n_{s}L_{OEO}}\left(1-P_{e}\right)\cdot k_{0}n\cdot \frac{2l_{GMM}}{\pi d+2l_{1}}\cdot k\cdot z_{1}\Delta B \end{array}$$

The variation of the magnetic field has been mapped to the frequency of the OEO oscillation signal, establishing a one-to-one relationship between the microwave domain and the magnetic field, as indicated by Equation (6). This allows for precise measurement of magnetic field variations by observing the OEO oscillation frequency. The dynamic range (DR) of the measurement is connected to the sensitivity of measurement $S_{B}$ and the free spectral range (FSR) of the OEO, which is represented as:

(7) $$DR=\frac{FSR}{S_{B}}=\frac{FSR}{\Delta f_{e}/B}=\frac{\pi \cdot \left(\pi d+2l_{1}\right)}{\left(1-P_{e}\right)\cdot k_{0}n\cdot l_{GMM}\cdot k\cdot z_{1}}$$

where $FSR=c/n_{s}L_{OEO}$ is the interval between the OEO oscillation modes. As per Equation (7), the DR is not contingent upon the length of the OEO loop. Nonetheless, it is associated with the length of the fiber encircling Region 1.

## 3. Results and Analysis

An experiment utilizing the setup shown in Figure 1 is conducted to demonstrate an OEO-based magnetic field sensing system. The TLS (NKT Photonics, Birkerød, Denmark, BASIK E15) is configured with an output power of 13.07 dBm and wavelength of 1550.591 nm. The MZM (JDS Uniphase, San Jose, CA, USA) has a half-wave voltage of 6.8 V and operates with a 3 dB bandwidth of 10 GHz. The PS-FBG is used as an optical bandpass filter, with a center wavelength of 1550.604 nm and a 3 dB bandwidth of 0.015 nm. The PD (Multiplex, NJ, USA, MTRX192L) exhibits a responsivity of 0.8 A/W and a 3 dB bandwidth of 10 GHz. Meanwhile, the EBPF (Dehui Communication Equipment Store, Jinan, China, Sample, BP1090-20) has a 3 dB bandwidth of 20 MHz and a central frequency of 1090 MHz. The EA (JDS Uniphase, San Jose, CA, USA, H301-1210), used for compensating loop loss, provides an amplification gain of 26 dB and features a 3 dB bandwidth of 10 GHz. The key sensing element of the magnetostrictive transducer is a rectangular block of the GMM (REMA-CN, Huizhou, China, TbDyFe), with dimensions of L×W×H:40×8×4 mm. The support arm, shaped as a semicircular plate with an outer diameter of 45 mm, is securely attached using UV glue. To improve response sensitivity, multiple turns of fiber are tightly coiled around it. Additionally, we assume that the fiber is subjected to uniform strain along its length, as it is wound around the magnetostrictive transducer.

**Figure 4:**
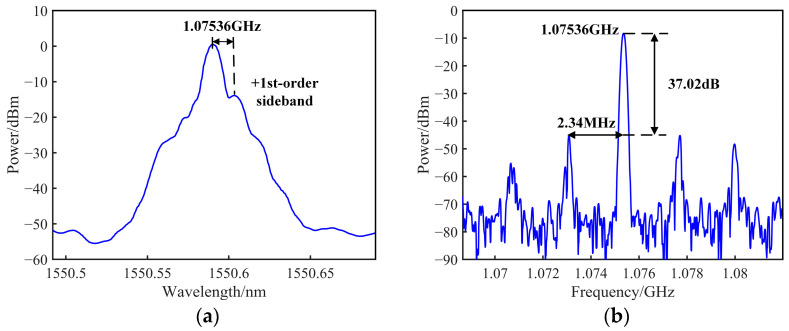
(**a**) Optical spectrum at the Pol output; (**b**) electrical spectrum of the OEO’s 1.07536 GHz oscillation signal.

The optical spectrum measured at the Pol output using an OSA (YOKOGAWA, Tokyo, Japan, ANDO, AQ6370D) is depicted in Figure 4a after establishing the OEO feedback loop. Due to the filtering effect of the PS-FBG, the −1st-order sideband is suppressed, while the optical carrier and +1st-order sideband are retained. The oscillation signal measured in the OEO loop is a beat signal, resulting from the interference between the optical carrier and the +1st-order sideband. Meanwhile, Figure 4b displays the electrical spectrum of the corresponding OEO oscillating signal captured using an ESA (Agilent Technologies, Santa Clara, CA, USA, Agilent N9010A). The OEO system produces a microwave signal centered at 1.07536 GHz, exhibiting a side mode suppression ratio (SMSR) of 37.02 dB. Furthermore, the FSR of the OEO is determined to be 2.34 MHz, which corresponds to an OEO loop length of 88 m. The observed center frequency and FSR of the OEO oscillating signal in the experiment align closely with the theoretically derived results, validating the accuracy of the theoretical model and experimental setup.

**Figure 5:**
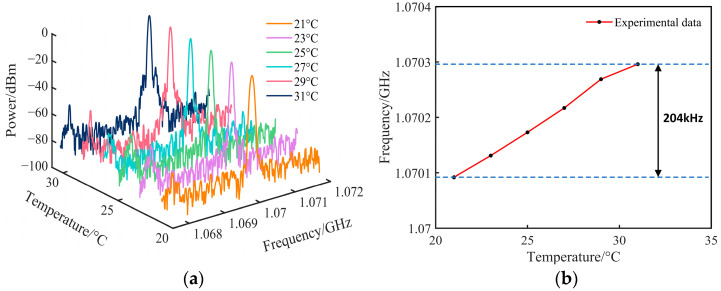
(**a**) Spectra of temperature stability testing for the sensing system; (**b**) variation of oscillation frequency with temperature.

To optimize fiber lengths $z_{2}$ and $z_{3}$ for temperature insensitivity, we conducted a temperature sensing experiment. The untreated fiber (Regions 2 and 3) and sensing component (Region 1) were both positioned on a glass plate inside a temperature-controlled chamber. According to the theoretical derivation of Equation (2) and the construction of the actual experimental environment, it was determined through testing that when $z_{2}$ and $z_{3}$ are 56 cm and 230 cm, respectively, (where $z_{1}=(4.5\pi +1)\times 7\;\mathrm{cm}\approx 106\;\mathrm{cm}$ is the total fiber length wound around the magnetostrictive transducer), the temperature effect is minimized. Figure 5a shows the electrical spectra of temperature stability testing. As depicted in Figure 5b, the total change in the center frequency of the OEO oscillation signal is 204 kHz as the temperature rises from 21 °C to 31 °C. This means that within a 10 °C variation span, the temperature-induced measurement error for the magnetic field is 0.03 mT, which is the result after temperature compensation. Once there is no temperature compensation, a change of 10 °C would lead to a frequency shift of over 210 MHz, according to Equation (1).

**Figure 6:**
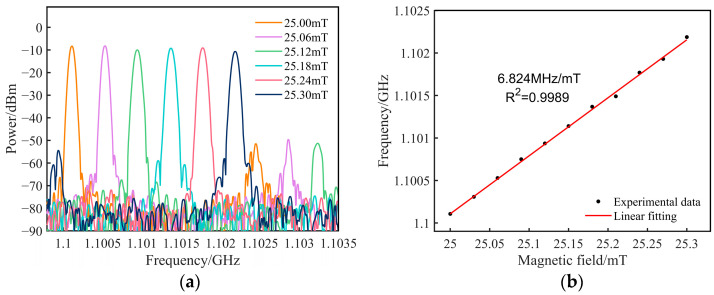
(**a**) Superposition spectrum of the oscillating signals as the magnetic field incrementally rises; (**b**) variation of oscillation frequency with magnetic field.

The sensing performance of the magnetic field sensor is evaluated. The untreated SMF and sensing component are placed within a solenoid, where the magnetic field intensity is influenced by variations in power supply current, and the direction of the magnetic field is parallel to the GMM. As depicted in Figure 6, when the magnetic field rises from 25.00 mT to 25.30 mT in steps of 0.06 mT, the OEO oscillating frequency linearly increases. Figure 6a shows the superposition spectrum of the oscillation signals as the magnetic field incrementally rises. The test data presented in Figure 6b are subjected to linear fitting, revealing a magnetic field sensitivity of 6.824 MHz/mT and a correlation coefficient (R^2^) of 0.9989. The frequency shift of this signal is directly proportional to the change in magnetic field, as demonstrated by the linear relationship shown in Figure 6b. This relationship is a key feature of the proposed sensor, enabling high-sensitivity magnetic field detection.

**Figure 7:**
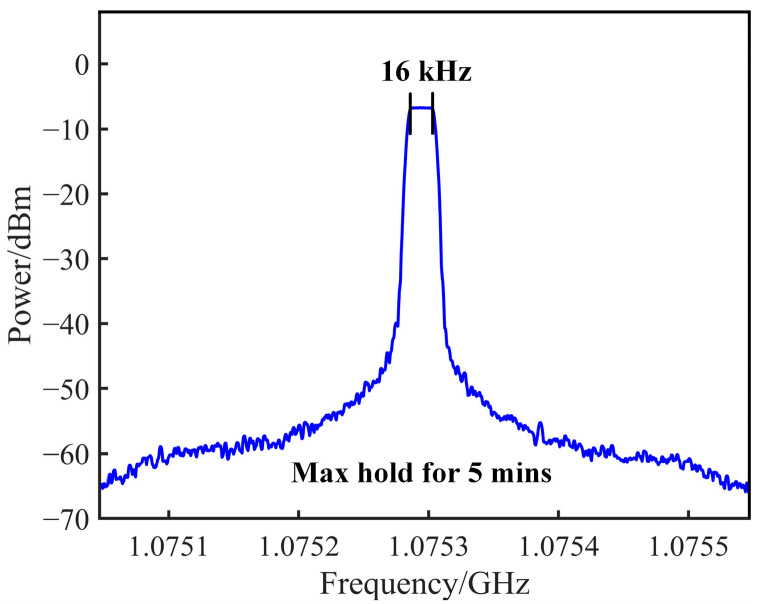
Frequency stability measurement for 5 min at “maxhold” mode.

We have also analyzed the sensing accuracy of the proposed OEO system through multiple experiments. Each experiment yielded consistent results, with minimal variation in measurement accuracy. In the experiments, the ESA is configured to operate in “maxhold” mode, covering a 500 kHz span, to measure and evaluate the frequency stability. The center frequency of the oscillating signal exhibits a shift of approximately 16 kHz over a span of 5 min, corresponding to a sensing accuracy of 0.002 mT, as depicted in Figure 7. The main factor contributing to this frequency shift is the random change of polarization components of light within the SMF, leading to phase difference variations between the x and y polarization components. Replacing SMF with polarization-maintaining fiber (PMF) in the optical path can significantly improve frequency stability.

Finally, the sensor’s DR and resolution are analyzed to evaluate its performance. Based on the experimental results and Equation (7), the DR is approximately ±0.17 mT (corresponding to a magnetostrictive phase shift of ±π). Theoretically, the resolution of the sensor is determined by the full-width at half-maximum (FWHM) of its main mode and its measurement sensitivity [23]. Experimental data indicate that the FWHM of the main mode is 7 kHz, yielding a calculated resolution of 0.001 mT. Increasing the number of the fiber turns around the magnetostrictive transducer can effectively enhance magnetic field sensitivity and measurement resolution by amplifying the strain-induced phase shift. However, this improvement comes at the cost of a reduced DR, as the additional turns of the optical fiber will cause the oscillating signal to reach the measurement threshold more rapidly, which is contingent upon the FSR of the OEO. Therefore, a careful trade-off between DR and resolution is essential to optimize the sensor’s performance for specific practical applications.

## 4. Conclusions

In summary, we have developed and experimentally validated a high-performance magnetic field sensor that exhibits remarkable temperature insensitivity, making it highly suitable for applications in environments with fluctuating thermal conditions. This sensor employs an MZI configuration with an integrated OEO demodulation system, which plays a pivotal role in mitigating temperature-induced crosstalk. By leveraging the OEO system to monitor the center frequency of the generated microwave signal, the sensor achieves precise magnetic field detection unaffected by temperature variations. The experimental results demonstrate an impressive magnetic field sensitivity of 6.824 MHz/mT and a measurement resolution of 1 µT, highlighting the system’s capability for high-precision detection. Furthermore, we have identified that increasing the number of the fiber turns around the magnetostrictive transducer can further enhance both the magnetic field sensitivity and resolution. However, this improvement comes at the cost of a potential reduction in the DR of the sensor, necessitating a careful balance between resolution and DR for specific application requirements. The integration of OEO with fiber-optic magnetic field sensing technology represents a significant advancement, enabling high-sensitivity magnetic field detection with robust temperature insensitivity. This innovation delivers exceptional performance in terms of resolution, accuracy, and reliability, making it a promising solution for a wide range of magnetic field sensing applications.

## Data Availability

The original contributions presented in this study are included in the article. Further inquiries can be directed to the corresponding author.
